# Colorectal cancer in a patient with intestinal schistosomiasis: a case report from Kilimanjaro Christian Medical Center Northern Zone Tanzania

**DOI:** 10.1186/s12957-017-1217-1

**Published:** 2017-08-02

**Authors:** Ayesiga M Herman, Alfred Kishe, Heri Babu, Hilary Shilanaiman, Murad Tarmohamed, Jay Lodhia, Patrick Amsi, Jeremia Pyuza, Alex Mremi, Amos Mwasamwaja, Mramba Nyindo, Kondo Chilonga, David Msuya

**Affiliations:** 1 Department of General Surgery, Kilimanjaro Christian Medical Centre, P.O Box 3010, Moshi, Tanzania; 2 Department of Pathology, Kilimanjaro Christian Medical Center, P. O Box 3010, Moshi, Tanzania; 3 Department of Endoscopy, Kilimanjaro Christian Medical Center, P. O Box 3010, Moshi, Tanzania; 4 Kilimanjaro Christian Medical University College, P. O Box 2240, Moshi, Tanzania

**Keywords:** Intestinal schistomoiasis, Colorectal cancer, *Schistosoma mansoni*, *Schistosoma japonicum*

## Abstract

**Background:**

Colorectal cancer associated with chronic intestinal schistosomiasis has been linked with the chronic inflammation as a result of schistosomal ova deposition in the submucosal layer of the intestine. Among all species *Schistosoma japonicum* has been more linked to development of colorectal cancer as compared to *Schistosoma mansoni* due to absence of population-based studies to support the association. Despite the weak evidence, some cases have been reported associating *S. mansoni* with development of colorectal cancer.

**Case Presentation:**

We report a patient who presented to us as a case of intestinal obstruction and found to have a constrictive lesion at the sigmoid colon at laparotomy, then later found to have colorectal cancer with deposited *S. mansoni* ova at histology.

**Conclusion:**

Given the known late complications of schistosomiasis, and as *S. mansoni* is endemic in some parts of Tanzania, epidemiological studies are recommended to shed more light on its association with colorectal cancer.

## Background

Colorectal cancer (CRC) is reported to have varying degrees of incidences worldwide, with its prevalence on the increase in the developing nations, mainly due to change of diet (a low fiber, high fat diet) and physical inactivity [1]. These factors are among the common universally known risk factors for colonic adenocarcinoma [2, 3] with some rare risk factors like schistosomal colitis being observed in Africa, Middle East, and South-East Asia [4]. Studies have highlighted an association between *Schistosoma japonicum* infections with adenocarcinoma of the colon while the association of *S. mansoni* with colorectal cancer remains with few supporting evidence [4–6]. We present the first documented case in Tanzania of a patient with intestinal schistosomiasis associated with colorectal cancer from Lower Moshi Tanzania (an area with irrigation scheme for rice fields and also with high prevalence of schistosomiasis) who presented to us as a case of intestinal obstruction and found to have adenocarcinoma of sigmoid colon and a deposited *S. mansoni* ova at histology after laparotomy.

## Case presentation

We present a 52-year-old male, a referral patient to Kilimanjaro Christian Medical Centre (KCMC) from a health center in Lower Moshi presenting with a 4 days history of abdominal pain, distension, and constipation. There were no other remarkable symptoms in the past, and no history of abdominal surgery.

On examination, he was fully conscious, with 37^o^ C body temperature, blood pressure 115/70 mmHg, pulse rate 92 beats per minute and oxygen saturation 94% in room air.

The abdomen was uniformly distended with mild tenderness. Bowel sounds were normal. On digital rectal examination, the patient had a normal anal verge and no mass palpable per rectum.

**Fig. 1:**
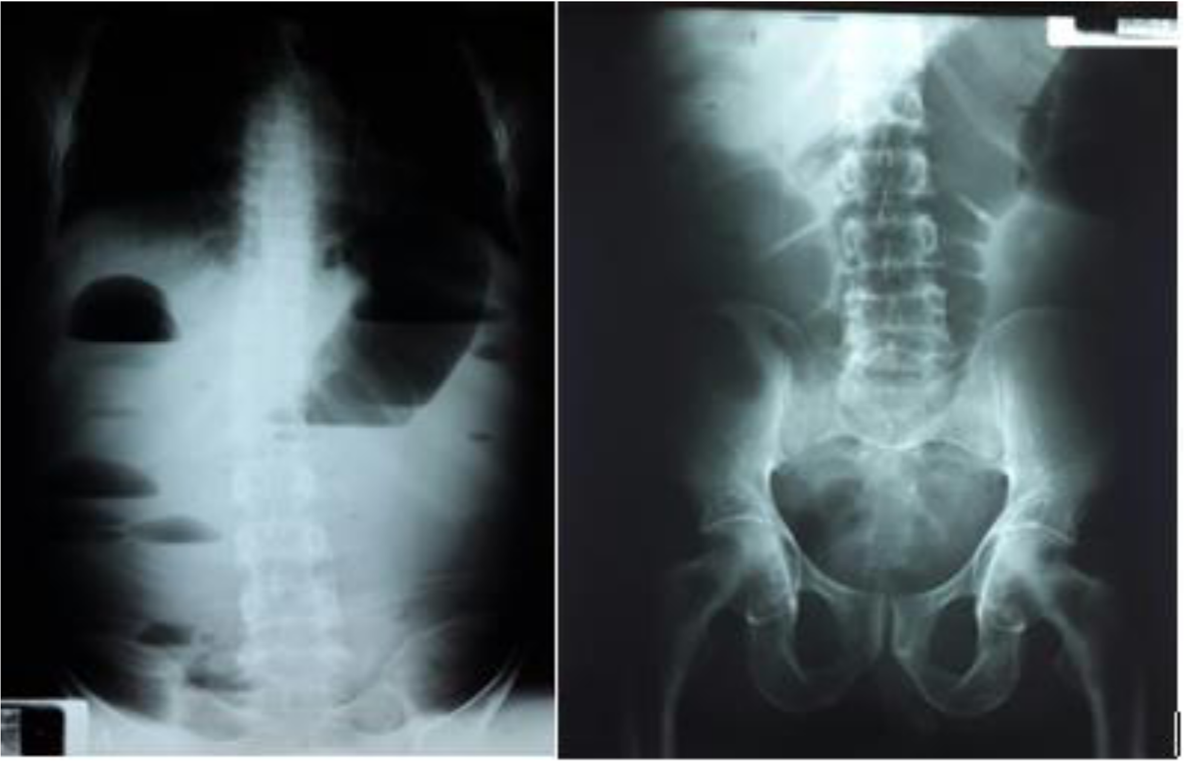
Plain abdominal X-rays showing multiple air fluid levels with dilated large bowel

Investigations were done included plain abdominal X-ray which showed dilated large bowel with multiple air fluid levels and empty rectum (Fig. 1).

Abdominal ultrasound found gaseous abdomen with a normal homogeneous liver texture.

The full blood count showed low levels of lymphocytes and platelets otherwise other blood cell parameters were normal with the hemoglobin of 14 g/dL and erythrocyte sedimentation rate of 1 mm/1 h.

The patient was blood group O positive with all renal function tests, electrolytes levels, and transaminases enzymes being with in the normal range.

He was diagnosed to have intestinal obstruction (large bowel obstruction) and a decision for emergency exploratory laparotomy was made.

**Fig. 2:**
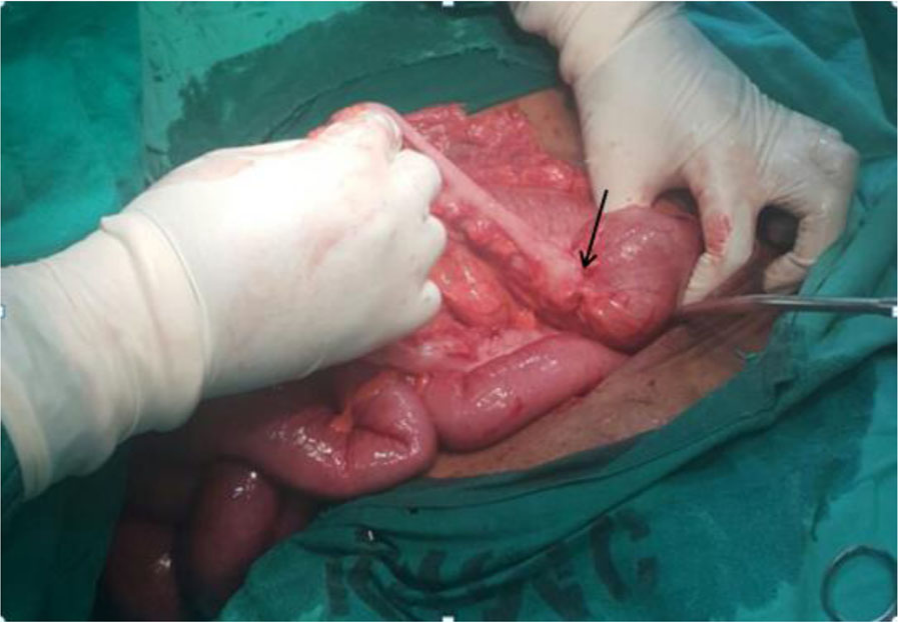
Constricted part at sigmoid colon at laparotomy

Intra operative findings were grossly distended ascending, transverse, and descending colon with a fibrotic like constricting lesion (Fig. 2 black arrow) at the proximal sigmoid colon and a collapse of the rest of the sigmoid colon. No lymph nodes were found in the mesocolon and other organs were normal (TNM stage cT3N0M0).

Sigmoidectomy together with the constricted lesion was done followed by a double barrel colostomy. The specimen was sent for histopathology.

The patient recovered well and was discharged to be followed up in the outpatient clinic for the histopathology results.

**Fig. 3:**
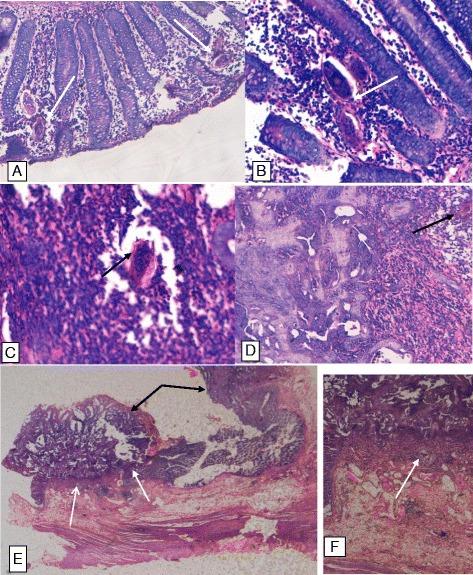
Histopathology of the colon with *Schistosoma mansoni* and adenocarcinoma of the colon. **a** and **b** deposited shcistosomal ova (*white arrows*) in the lamina propria; **c** Schistosoma mansoni ova (*black arrow*) with with lateral spine and sorrounding chronic granulomatous inflamation; **d** Schistosoma ova (*black arrow*) in close proximity with colonic adenocarcinoma; **e** Cross section of the colonic wall showing areas with a poorly differentiated adenocarcinona (*black arrows*) and diposited schistosoma ova (*white arrows*) in close proximity with the adenocarcinoma; **f** X 10 magnification of figure **e** showing schistosoma ova with sorroundig granulomatus inflamation in close proximity with adenocarcinoma of colon

The histology results showed a non-caseating granuloma, containing a centrally located *S. mansoni* ovum. Another area of the histopathology specimen had colonic tissue with complex glandular proliferation with hyper chromatic nuclei and exhibiting cribriform pattern invading the lamina propria with a conclusion of a poorly differentiated adenocarcinoma pT2N0M0. (Fig. 3).

Histological diagnosis of adenocarcinoma of the colon and intestinal schistosomiasis was reached.

At 6 weeks post the initial surgery colonoscopy performed for the proximal and distal loops was found to be normal. A left hemi colectomy was done, and the patient was referred to the oncology unit.

## Discussion

Intestinal schistosomiasis has been linked to development of colorectal cancers with a number of documented cases reported in some parts of Africa and Asia [4–7]. Both *S. mansoni* and *S. japonicum* cause intestinal schistomiasis. However, *S. japonicum* has been reported to be more associated with colorectal neoplastic changes than *S. mansoni* [6–8]. However, there have been reported cases of colorectal cancer attributed to *S. mansoni* even though most of the conclusions from these reports recommend further studies to establish the association [5, 6, 8–10].

Intestinal schistosomiasis causes a series of changes similar to those brought about by inflammatory bowel diseases [10–13]. Vennervald and Polman (2009) highlighted chronic inflammation as a key feature in development of cancerous lesions in chronic schistosomiasis [14]. This is due to the triggering of a granulomatous response post submucosal deposition of schistosoma ova, which may cause pseudopolyps formation and mucosal ulceration leading to dysplasia and later neoplastic changes [5, 7, 10, 12]. Furthermore, chronic inflammation is believed to cause microsatellite instability leading to damaged DNA repair mechanism; a key factor to neoplastic changes [14, 15]. A study conducted by Soliman et al. [16] on molecular pathology for colorectal carcinoma contrasting Egyptian and Western patients found high rates of microsatellite instability and K-ras mutation in Egyptian patients to be associated with schistosoma infection.

In Tanzania, colorectal cancer (CRC) incidences have been on the increase. A study on the pattern and distribution of CRC reported six times increase in incidences in the last decade [17]. However, there is limited information on intestinal schistosomiasis linked to development of colorectal cancer. In contrast, there are reported cases on *S. hematobium* with development of bladder cancer and prostate cancer [18, 19]. In addition, *S. mansoni* has been widely associated with portal hypertension with most patients presenting with upper gastrointestinal bleeding as a consequence of periportal fibrosis [20]. With regards to the association between CRC and parasitic infections, the contribution of schistosoma infections to colon and rectal neoplasm has not been quantified despite the endemicity in some parts of the country. It is important to note that in the areas where schistosomiasis is endemic, it would be an added risk factor which some authors have termed as an independent risk factor to development of CRC that needs to be addressed [10].

The prevalence of schistosomiasis in Tanzania has been reported to increase from 19% in 1977 to 51.5% in 2012 [21]. Given the prevailing morbidity, this has a major implication on the late complications to the community, colorectal neoplasms being among them as it has been associated with colonic schistosomiasis [10]. According to global health burden of infection in association to cancers report of the year 2002, 17.8% of all cancers were attributed to infections where by schistosomes contributed 0.1% to this burden [22]. Though schistosoma was labeled to have a minimal contribution to the cancer burden in the report by Parkin [22], the magnitude in endemic areas needs to be further studied. This will potentiate the induction of preventive programs and hence reduce late complications.

There are and have been national wide programs to treat and control schistosoma infections as highlighted by Mazigo et al. [21]. However, dealing with the complications of the infections poses a great challenge to the health sector given the high prevalence of the disease in Tanzania. Public health education on complications and their presentations is recommended for groups at risk. Furthermore, especially with regards to neoplasms, screening services may play an important role to capture individuals affected in endemic regions at early stages. We believe this should go hand in hand with the current programs that have been launched national wide to help individuals who are already affected by schistosomiasis.

## Conclusion

There are case reports that have linked *S. mansoni* with colorectal neoplasm in different parts of the globe with very scarce information on this association being reported from Tanzania. Given that *S. mansoni* is endemic in some parts of the country, we recommend further epidemiological studies that will help in establishing the association.

## Abbreviations

CRC: Colorectal cancer
TNM: Tumor, nodes, and metastasis
